# 

**Published:** 1858-02

**Authors:** 


					﻿PUBLISHED MONTHLY.
VOL. L]
[No. 2.
CHIC4QO
MEDICAL JOURNAL
■DITED BT
INT. S. DAVIS,	T).,
AND
W. EC, BTTFORD, ZMC.JD.
FEBRUARY, 1858.
BARNET & CLARKE, BOOK AND JOB PRINTERS, “CHRONOTYPE” OFFICE,
189 LAKE STREET, CORNER OF WELLS.
P. O. Box 2298,]
[Chicago, Bl.
AT $2 FEB ANNUM, IN ADVANCE.
				

